# The role of plasminogen activator inhibitor-2 in pneumococcal meningitis

**DOI:** 10.1186/s40478-022-01461-1

**Published:** 2022-10-29

**Authors:** Nina C. Teske, Joo-Yeon Engelen-Lee, Susanne Dyckhoff-Shen, Hans-Walter Pfister, Matthias Klein, Diederik van de Beek, Carsten K. Kirschning, Uwe Koedel, Matthijs C. Brouwer

**Affiliations:** 1 Laboratory of Neuroinfectious Diseases, Department of Neurology, Klinikum Großhadern, Ludwig-Maximilians University, Munich, Germany; 2 Department of Neurology, Amsterdam UMC, University of Amsterdam, Amsterdam Neuroscience, PO Box 22660, 1100 DD Amsterdam, The Netherlands; 3 European Society for Clinical Microbiology and Infectious Diseases (ESCMID) Study Group for Infections of the Brain (ESGIB), Basel, Switzerland; 4 Institute of Medical Microbiology, University of Duisburg-Essen, Essen, Germany

**Keywords:** Pneumococcal meningitis, Plasminogen activator inhibitor-2, SERPINB2

## Abstract

Pneumococcal meningitis is associated with dysregulation of the coagulation cascade. Previously, we detected upregulation of cerebral plasminogen activator inhibitor-2 (PAI-2) mRNA expression during pneumococcal meningitis. Diverse functions have been ascribed to PAI-2, but its role remains unclear. We analyzed the function of SERPINB2 (coding for PAI-2) in patients with bacterial meningitis, in a well-established pneumococcal meningitis mouse model, using *Serpinb2* knockout mice, and in vitro in wt and PAI-2-deficient bone marrow-derived macrophages (BMDMs). We measured PAI-2 in cerebrospinal fluid of patients, and performed functional, histopathological, protein and mRNA expression analyses in vivo and in vitro. We found a substantial increase of PAI-2 concentration in CSF of patients with pneumococcal meningitis, and up-regulation and increased release of PAI-2 in mice. PAI-2 deficiency was associated with increased mortality in murine pneumococcal meningitis and cerebral hemorrhages. *Serpinb2*^*−/*−^ mice exhibited increased C5a levels, but decreased IL-10 levels in the brain during pneumococcal infection. Our in vitro experiments confirmed increased expression and release of PAI-2 by wt BMDM and decreased IL-10 liberation by PAI-2-deficient BMDM upon pneumococcal challenge. Our data show that PAI-2 is elevated during in pneumococcal meningitis in humans and mice. PAI-2 deficiency causes an inflammatory imbalance, resulting in increased brain pathology and mortality.

## Introduction

Pneumococcal meningitis (PM) is the most common form of bacterial meningitis and is associated with high mortality and risk of neurological sequelae [5, 6]. Cerebrovascular complications are an important cause of poor outcome and death [34, 38]. Activation of the coagulation cascade and inhibition of fibrinolysis have been shown to be associated with the risk of cerebral infarction in meningitis [7, 33, 43, 44]. A direct interaction by pneumococcal virulence factors with coagulation or fibrinolysis factors has been suggested, although it is difficult to dissect whether this is independent of the inflammatory response [41].

Plasminogen activator inhibitor-1 (PAI-1) is an important regulator of fibrinolysis by inhibiting tissue plasminogen activator (tPA) and urokinase (uPA) mediated proteolytic degradation of fibrin clots [23]. In a study on the role of PAI-1 in PM we showed that the functional genetic variation rs1799889 in *SERPINE1* (coding for PAI-1) influenced the risk of cerebral infarction, hemorrhage, poor disease outcome and mortality in patients [7]. Subsequently, in a mouse model of PM we demonstrated that PAI-1-deficiency resulted in higher mortality and increased cerebral hemorrhages, suggesting a protective role of PAI-1 [7].

Analogous to PAI-1, PAI-2 (also known as SERPINB2) is classically viewed as an inhibitor of extracellular uPA and tPA, even if PAI-2 is slower than PAI-1 at inhibiting these proteases (in vitro) [10, 15]. However, it is unclear what its key function is, given that the vast majority of PAI-2 is located intracellularly in the cytoplasm. A broad range of activities has been ascribed to PAI-2, such as regulation of monocyte proliferation and differentiation [46], priming interferon α/β responses [3], inhibition of annexin-1 cleavage [13], interleukin 1β processing [17] and also apoptosis [14]. Despite over 1000 publications on PAI-2, no consensus view on its (patho)physiological function has been reached, which has led to labels like “the undecided Serpin” [29].

Some studies using PAI-2-deficient mice showed that PAI-2 can modulate Th1/Th2 immune response, implicating a role in adaptive immunity [27, 36, 37, 47]. Our knowledge of the PAI-2 functions in innate immunity is limited. PAI-2 antigen could be detected in the plasma of sepsis patients and was associated with mortality [21, 35]. In PAI-2-deficient mice however, no differences to wild type (wt) mice in survival were found after challenge with endotoxin or cecal ligation and puncture [11]. An in vitro model of monocytic cells showed stimulation with *B. burgdorferi* induced production and secretion of PAI-2. This resulted in significantly diminished uPA-dependent invasion by monocytic cells, proposing a role of PAI-2 in leukocyte migration [20]. Studies focusing on the regulation of PAI-2 expression after stimulation with bacteria and/or bacterial components showed significant up-regulation of PAI-2 expression in mononuclear cell and/or macrophages upon exposure [4, 20, 39, 40]. In a mouse model of PM, we observed a strong upregulation of PAI-2 mRNA expression in brains of infected mice [33]. So, it seems apparent that PAI-2 is formed during bacterial infections, but the specific role PAI-2 plays in infections remains unclear.

To determine the role of PAI-2 during PM we analyzed PAI-2 concentrations in cerebrospinal fluid of pneumococcal meningitis patients and immunohistochemically stained PAI-2 in brains of patients who died of pneumococcal meningitis. Subsequently, we compared the disease course in wt versus PAI-2-deficient mice and the response of wt versus PAI-2-deficient BMDM upon pneumococcal challenge.

## Methods

### Dutch bacterial meningitis cohort

In a nationwide prospective cohort study, we included patients with community-acquired bacterial meningitis with an age of 16 years or older with positive CSF cultures who were identified by The Netherlands Reference Laboratory for Bacterial Meningitis (NRLBM) from March 2006 to October 2011 [5, 25]. The NRLBM provided the names of the hospitals where patients with bacterial meningitis had been admitted 2–6 days previously. The treating physician was contacted, and written informed consent was obtained from all participating patients or their legally authorized representatives, and controls. Methods of the study have been described in detail previously [5]. The study was approved by the Medical Ethics Committee of the Academic Medical Center, Amsterdam, The Netherlands.

### CSF PAI-2 analysis

CSF of patients with PM (*n* = 268) was obtained from the diagnostic lumbar puncture and subsequently stored at − 80°C. Control CSF samples (*n* = 14) were obtained from patients with benign thunderclap headache in whom a lumbar puncture was performed to rule out subarachnoid hemorrhage. CSF PAI-2 levels were determined using the Millipore Luminex kit according to the manufacturer’s instructions (Merck Millipore, MA, USA). We performed association analysis between the CSF concentrations of PAI-2 on admission and the clinical outcome (as measured by the Glasgow outcome scale, GOS) as well as the occurrence of cerebrovascular complications (comprising cerebral infarction and hemorrhages) in patients with pneumococcal meningitis. An unfavourable outcome was defined as a GOS score of 1–4, and a favorable outcome was defined as a score of 5. Cerebrovascular infarction and hemorrhages were defined as focal neurologic signs on admission or during the course of the disease with consistent findings on cranial computed tomography or magnetic resonance imaging.

### PAI-2 expression of human meningitis cases

Brain sections were obtained from patients included in the Dutch bacterial meningitis cohort. The brains were macroscopically examined, followed by formalin fixation. The sampled cut-up blocks were then embedded in paraffin, cut and stored. Brain sections were stained with primary antibodies directed against PAI-2 (Bio-Connect, Netherlands, dilution 1:100), the respective secondary antibody and chromogen. After counterstaining with hematoxylin solution, tissue sections were examined using a Carl Zeiss Axioskop light microscope.

### Animal pneumococcal meningitis model

We used a well-characterized mouse model of PM [22]. Briefly, mice were weighed, scored clinically, and the temperature was taken. Clinical scoring comprised a beam walk test, a postural reflex test, presence/absence of pilo-erection, reduced vigilance, and/or seizures. The maximum clinical score was 13, indicating moribund mice that had to be euthanized due to ethical considerations, whereas a score of 0 defined uninfected healthy mice. After initial clinical evaluation, bacterial meningitis was induced by intracisternal injection of 15 µl of 10^7^ colony forming units (cfu) per ml *Streptococcus pneumoniae* type 2 (D39 strain, kindly provided by Prof. Sven Hammerschmidt, University of Greifswald, Germany) under short-term anesthesia with isoflurane. To assess the role of PAI-2 in the development of meningitis, infected mice were observed for 24 h. To get insight into PAI-2 function in more advanced disease, mice were treated with ceftriaxone at 24 h to prevent death from overwhelming infection and assessed at 48 h post infection. At the end of each experiment, animals were clinically examined again. Thereafter, mice were anaesthetized with ketamine/xylazine, and a catheter was placed into the cisterna magna to obtain cerebrospinal fluid (CSF) samples for white blood cell (WBC) counts. Subsequently, animals were perfused transcardially with ice cold phosphate buffered saline (PBS). The cerebellum was dissected and homogenized in 1 ml sterile PBS for determination of bacterial titers. Cerebellar homogenates were diluted serially, plated on blood agar plates, and cultured for 24 h before colony forming units (CFU) were counted. Intracerebral hemorrhages were determined in frozen mouse brains, which were cut in a frontal plane into 10 μm thick sections. From the anterior parts of the lateral ventricles, ten serial sections were photographed with a digital camera at 0.3 mm intervals throughout the ventricle system. Hemorrhagic spots were counted, and – in selected experiments—the bleeding area was measured.

### Measurement of cytokine expression

In mice surviving up to the 48-h time-point, concentrations of C5a, IL-10, and IL-1β in homogenized brains were determined by ELISA (R&D Systems, Germany), according to the manufacturers’ instructions. Cytokine concentrations were given as amount of cytokine per mg brain protein.

### Experimental groups in the mouse model

The following experimental groups were investigated: (1) male C57BL6 wt mice injected intracisternally with 15 µl of PBS (so-called controls; *n* = 4 investigated 24 h after infection and *n* = 6 investigated 48 h after infection, respectively); (2) wt mice injected intracisternally with 15 µl of *S. pneumoniae* (*n* = 9 investigated 24 h after infection and *n* = 8 investigated 48 h after infection, respectively); (3) *Serpinb2*-deficient mice injected intracisternally with 15 µl *S. pneumoniae* (*Serpinb2*^−/−^, backcrossed for more than 10 generations onto the C57BL6 background, *n* = 9 investigated 24 h after infection and *n* = 12 investigated 48 h after infection, respectively). Supplemental experimental groups were carried out to analyze PAI-2 protein concentrations in murine CSF and plasma over the course of the disease, namely uninfected controls (*n* = 5), infected wt mice investigated 24 and 48 h after infection, respectively (*n* = 12 in each group).

### Immunohistochemical analysis

Brains from control and meningitic wt and *Serpinb2*-deficient mice were fixed in 10% formalin, embedded in paraffin, and cut into 7 µm sections. Brain sections were stained with primary antibodies directed against PAI-2 (ab137588, Abcam, dilution 1:100) and MRP-14, a marker for neutrophils (Invitrogen Antibodies, dilution 1:2000), the respective secondary antibodies and chromogens. After counterstaining with hematoxylin solution, tissue sections were examined using an Olympus BX-51 light microscope and images captured with a cooled Moticam 5000 video camera connected to a PC.

### Cell culture experiments with BMDM

Bone marrow–derived macrophages (BMDMs) were prepared from bone marrow cells obtained from femurs as described previously [22]. PAI-2-deficient and wt BMDM were exposed to increasing concentrations of the serotype 2 pneumococcal strain D39 (up to 10^6^ cfu/ml; that corresponds to MOI = 10). Four h after infection, BMDM were treated with ceftriaxone (100 µg/ml). Again 20 h later, medium samples were withdrawn and analyzed for cell viability using a commercially available LDH assay (Enzo Life Sciences) and for the presence of IL-10, IL-6, IL-1β (using ELISA kits from R&D Systems), and PAI-2 (using an ELISA Kit from FineTest®). We also isolated RNA from both infected and control PAI-2-deficient and wt BMDM and assessed the expression of the macrophage surface markers CX_3_CR1, ITGAM (CD11b), MRC1 (CD206), and CD36, of the macrophage phenotype markers iNOS and ARG1, as well as of PAI-2 by RT-PCR (using commercially available PrimePCR™ primer pairs from Biorad).

### Statistical analysis

Statistical tests were performed using GraphPad Prism and SPSS software. The principal statistical test was a Mann–Whitney U test for continuous variables, for grouped analyses a two-way ANOVA with Tukey’s multiple comparisons test or a log-rank test (Mantel) for survival. For correlation analysis we used spearman’s rank correlation coefficient. Differences were considered significant at *P* < 0.05. Patient data are displayed as median ± interquartile ranges (IQR), mouse data as mean ± standard deviation.

### Study approval

The animal experiments were approved by the animal ethic committee of the government of Upper Bavaria, Germany (AZ 211–2531-67/99 und -47/08).

## Results

### PAI-2 is released into the CSF and blood during pneumococcal meningitis

**Fig. 1:**
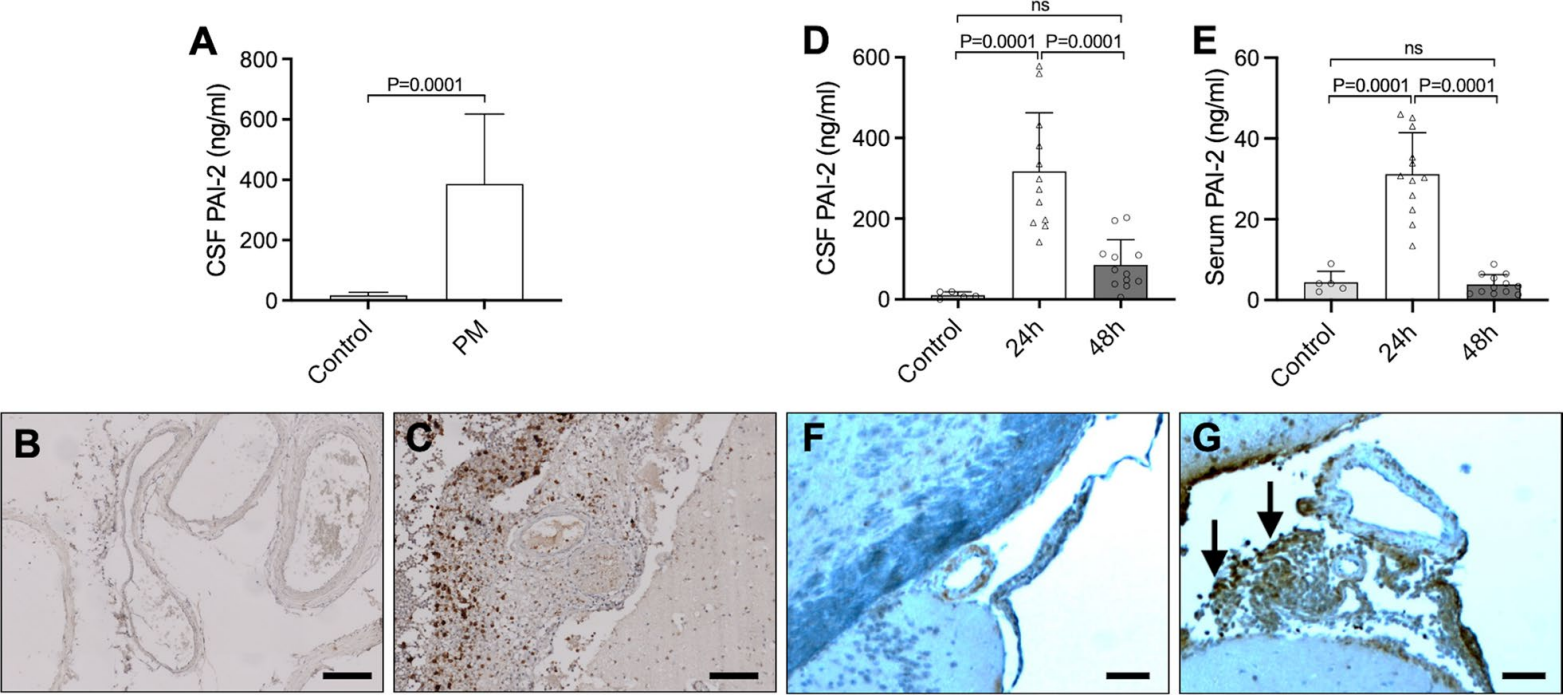
CSF PAI-2 levels (**a**) of patients included in the Dutch bacterial meningitis cohort with PM. Immunohistochemical analysis with PAI-2 of human brain sections obtained from uninfected control cases (**b**) and PM cases (**c**). Murine CSF (**d**) and plasma (**e**) PAI-2 levels 24 and 48 h after induction of PM. Immunohistochemical analysis with PAI-2 of murine brain sections obtained from uninfected control cases (**f**) and PM cases (**g**). The sections were stained with a PAI-2 antibody and counterstained with hematoxylin–eosin. Both human and murine immunohistochemical examination showed strong PAI-2 staining in the inflammatory infiltrate in the leptomeningeal space. The scale bar indicates 100 µm in length

We measured PAI-2 levels in CSF samples from 268 patients with PM. The median level of PAI-2 was 387 ng/ml (Interquartile range [IQR] 197—618), which was higher than CSF PAI-2 concentrations in 14 controls (median 18 ng/ml; IQR 0.3–27; *p* < 0.001) (Fig. 1a). CSF PAI-2 concentrations on admission were significantly higher in patients with an unfavourable outcome of pneumococcal meningitis (median 520 ng/ml, IQR 336–796 vs. 297 ng/ml, IQR 175–471 in patients with a favorable outcome; *p* < 0.001). CSF PAI-2 levels were also higher in patients who suffered cerebrovascular complications compared to those who did not (median 487 ng/mg IQR 331–749 vs. 370 ng/mg, IQR 193–542, P = 0.02). CSF PAI-2 was significantly correlated to CSF protein concentration (r = 0.63, *p* < 0.001). Immunohistochemical investigations showed strong PAI-2 staining in the inflammatory infiltrate in the leptomeningeal space (Fig. 1b, c).

Subsequently, we investigated whether the findings in patients with PM correspond to those in the mouse model. Previously, we observed a 15-fold up-regulation in PAI-2 mRNA expression in the brain of meningitis mice 24 h after pneumococcal infection compared to uninfected control mice [33]. Consistent with this, we found a marked increase in PAI-2 protein levels in CSF and plasma samples collected at 24 h after pneumococcal inoculation (median 285 ng/ml CSF PAI-2; IQR 192 – 419 versus median 8 ng/ml CSF PAI-2; IQR 3–19; *p* < 0.0001) (Fig. 1d, e). At 48 h CSF PAI-2 levels were not significantly different. Comparable to the patient data, the immunohistochemical examination showed PAI-2 positive cells particularly in the leukocyte infiltrate in the leptomeningeal space (Fig. 1f, g) and the ventricular system.

Taken together, we found a substantial up-regulation and release of PAI-2 in patients and mice with PM.

### PAI-2 deficiency is associated with worse outcome of murine pneumococcal meningitis

**Fig. 2:**
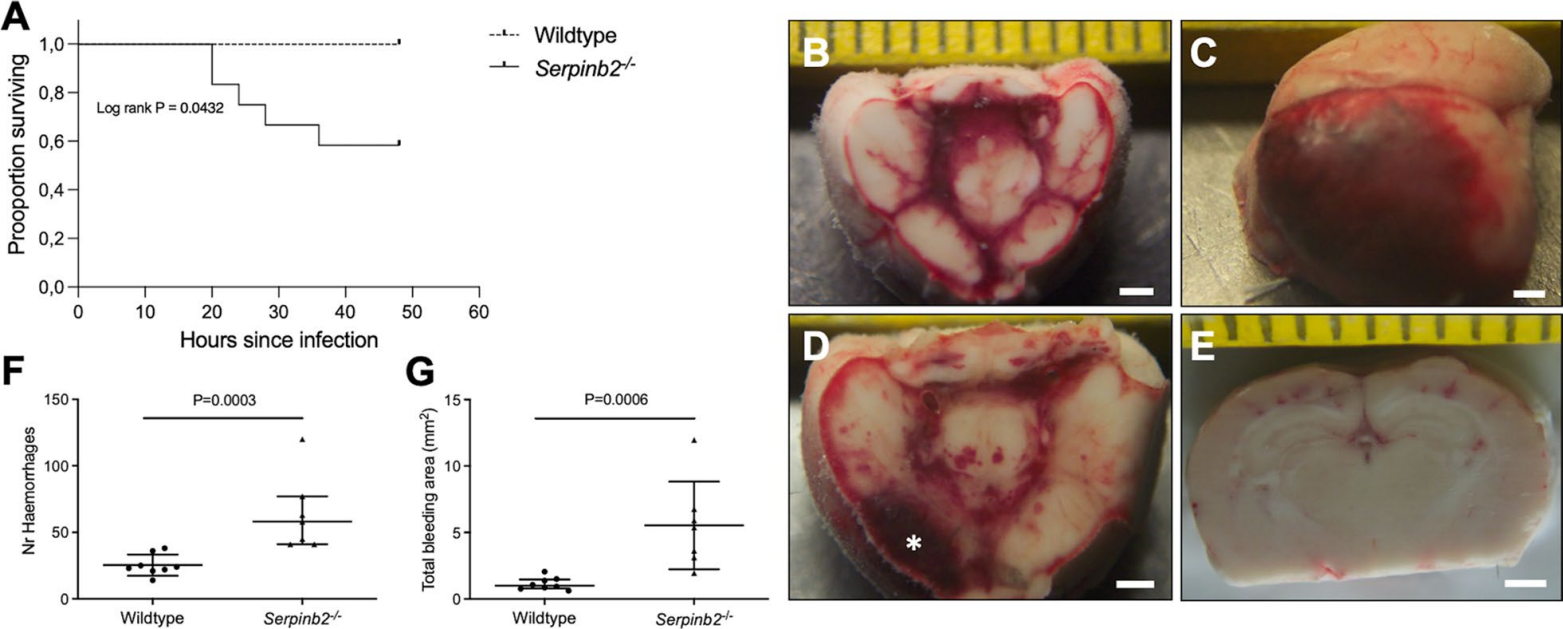
Kaplan Meier survival curve showing increased mortality in PAI-2-deficient (*Serpinb2*^−/−^) mice (**a**). Macroscopical images of PAI-2-deficient mouse brains (**b-e**). Basal view of the brain cut through the mesencephalon showing subarachnoidal bleeding (**b**). Left frontal view of the brain showing subdural hematoma over the left hemisphere (**c**). Basal view of the brain showing subarachnoidal and intracerebral hematoma (asterisk) (**d**). Multiple small cortical hemorrhages (**e**). Number of hemorrhages (**f**) and total bleeding areas (**g**) 48 h after pneumococcal inoculation in wt and PAI-2-deficient mice. The scale bar indicates 1 mm in length

**Fig. 3:**
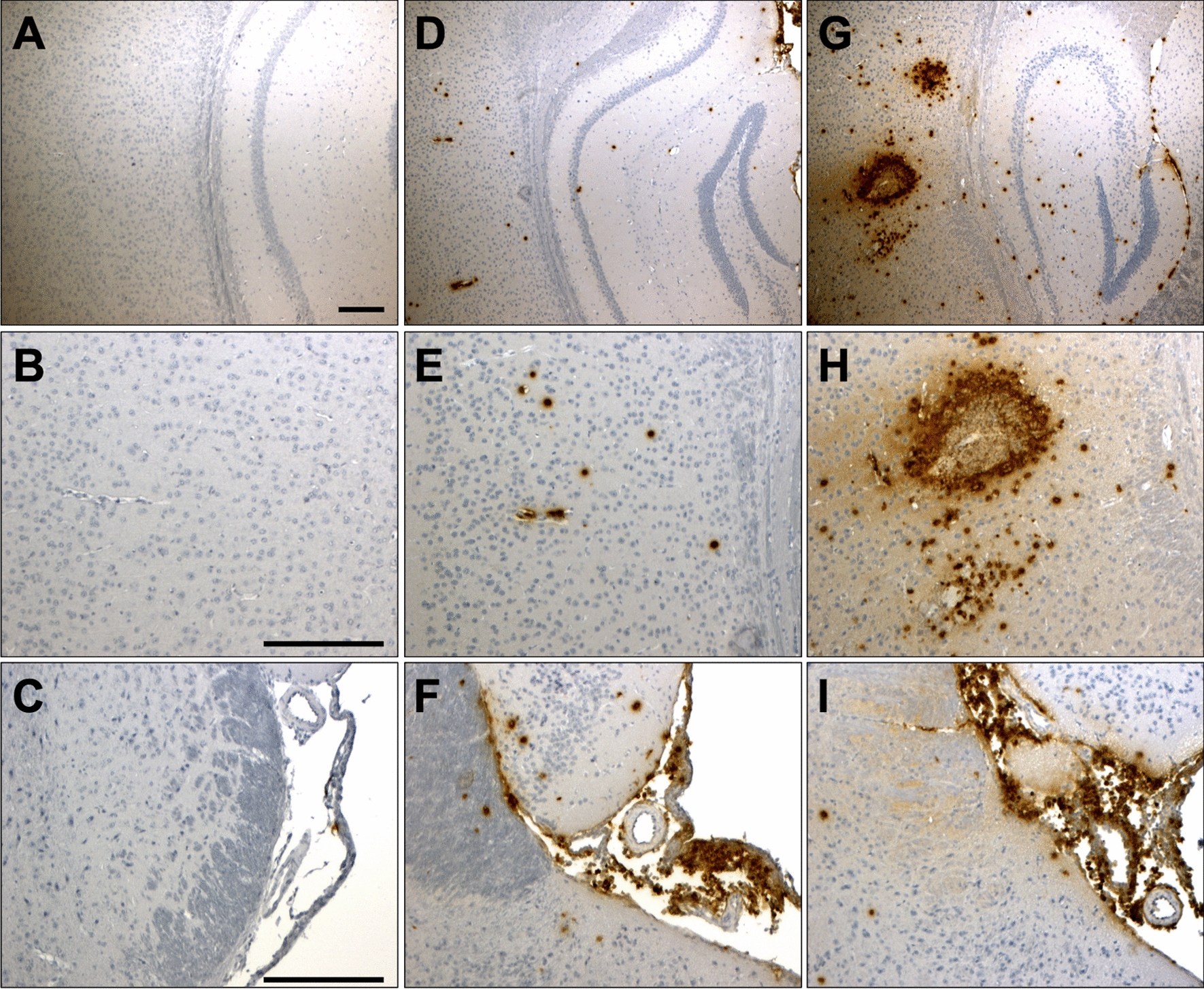
Immunohistochemical analysis with MRP-14 of brain sections obtained from non-infected wt mice (**a–c**), *S. pneumoniae-*infected wt mice (**d–f**) and *S. pneumoniae-*infected *Serpinb2*-deficient mice (**g–i**). Brain sections were obtained at 48 h after intracisternal application of PBS or *S. pneumoniae* (3 mice per group). The sections were stained with anti-murine MRP-14 antibody and counterstained with hematoxylin–eosin. Mice lacking *Serpinb2* showed enhanced MRP-14 staining in the brain parenchyma (**g**, **h**) and to a lesser degree also in infiltrates in the leptomeningeal space (**i**), compared to wt mice subjected to PM (**d–f**). The scale bar indicates 200 µm in length

To get better insight into the functional role of PAI-2 in PM, we compared the disease phenotype of PAI-2-deficient mice (*Serpinb2*^−/−^) to that of wt mice. In a first series of experiments, mice were examined 24 h after intracisternal inoculation of *S. pneumoniae*. At this time point, *Serpinb2*^−/−^mice showed signs of the disease that were identical to those of wt mice. There were no differences between groups in the numbers of CSF leukocytes and cerebral hemorrhages as well as in pneumococcal titers in the brain and blood. Consequently, PAI-2 does not seem to play a critical role in the initial stages of meningitis. Different results emerged when PAI-2-deficient mice were evaluated at advanced stages of the disease, 48 h after infection. In these experiments, mice were treated with ceftriaxone after 24 h comparable to a clinical situation. PAI-2 deficiency did not affect pneumococcal killing by ceftriaxone, CSF pleocytosis and clinical score values of surviving mice. Five of 12 PAI-2-deficient mice (41%) died before the end of the experiment, compared to none of the wt mice (Log-rank *P* = 0.04; Fig. 2a). Upon opening of the skull intracerebral (*n* = 1), subarachnoidal (*n* = 3) and subdural hemorrhages (*n* = 1) were detectable in 4 of 12 *Serpinb2*^*−/−*^ mice (33%) compared to none of the wt mice (Fig. 2b–e). Pathological brain examination revealed a greater number of hemorrhagic spots and larger total bleeding areas in PAI-2-deficient mice as compared to wt mice (mean number of hemorrhagic spots 63.6 *Serpinb2*^*−/−*^ vs. 25.4 wt, *p* < 0.001; mean total bleeding area 5.53 mm^2^ *Serpinb2*^*−/−*^ vs. 1.15 mm^2^ wt, *p* < 0.001; Fig. 2f, g). The increase in cerebral hemorrhages was accompanied with a more pronounced immune reactivity against MRP-14, a known marker of neutrophils, in infected *Serpinb2*^−/−^ mice compared to infected wt mice (Fig. 3). Thus, a higher level of neutrophilic infiltration around parenchymal vessels may contribute to the increase in cerebral hemorrhages in PAI-2-deficient mice with PM.

### *Serpinb2*^*−/−*^* mice exhibited increased C5a levels, but decreased IL-10 levels in the brain during pneumococcal infection*

**Fig. 4:**
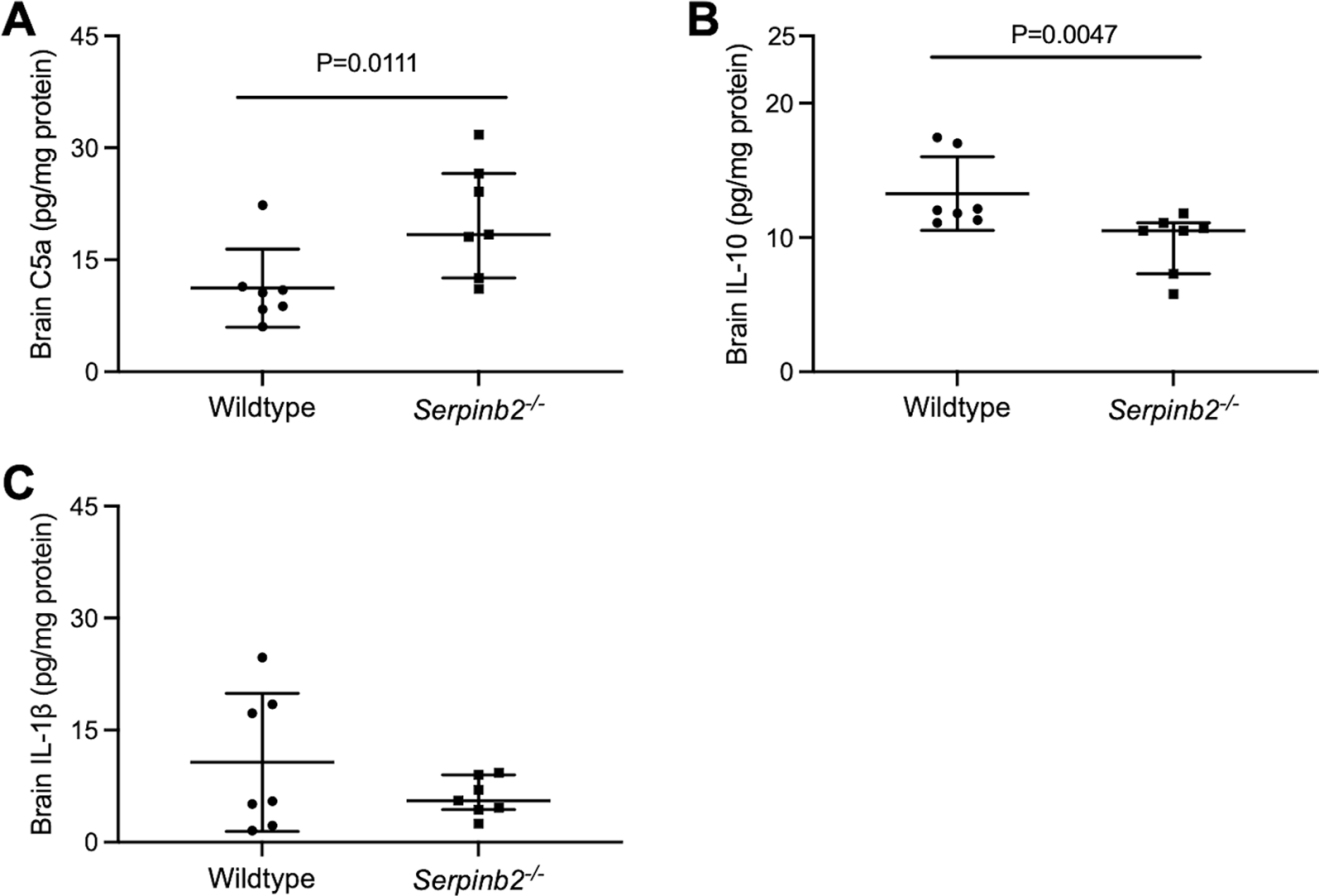
Brain C5a (**a**), IL-10 (**b**), IL-1β (**c**) determined in homogenized brain of infected mice surviving up to the 48-h time point. Mice lacking *Serpinb2* had significantly higher C5a concentrations and lower IL-10 levels compared to wt mice

PAI-2 was previously reported to suppress IL-1β production by controlling caspase-1 activation [9, 17]. In addition, PAI-2 deficiency can lead to increased uPA/tPA-dependent plasmin activity, which is capable of converting C5 into its active form C5a, thus resulting in elevated C5a concentrations [2, 12]. Both, IL-1β and C5a are known mediators of hyper-inflammation and vascular damage in murine PM [42]. This motivated us to determine C5a and IL-1ß concentrations in brains of infected wt and PAI-2-deficient mice and also measure brain levels of IL-10, a key anti-inflammatory cytokine in PM [24, 45, 48]. *Serpinb2*^*−/−*^ with PM had higher C5a concentrations (Fig. 4a), but lower IL-10 levels (Fig. 4b). Brain IL-1β levels were similar in both mouse strains (Fig. 4c). These data suggest dysregulated inflammation as a possible contributing factor of the aggravated brain pathology of PAI-2-deficient mice.

### PAI-2 deficiency led to decreased IL-10 release by BMDM upon pneumococcal infection

**Fig. 5:**
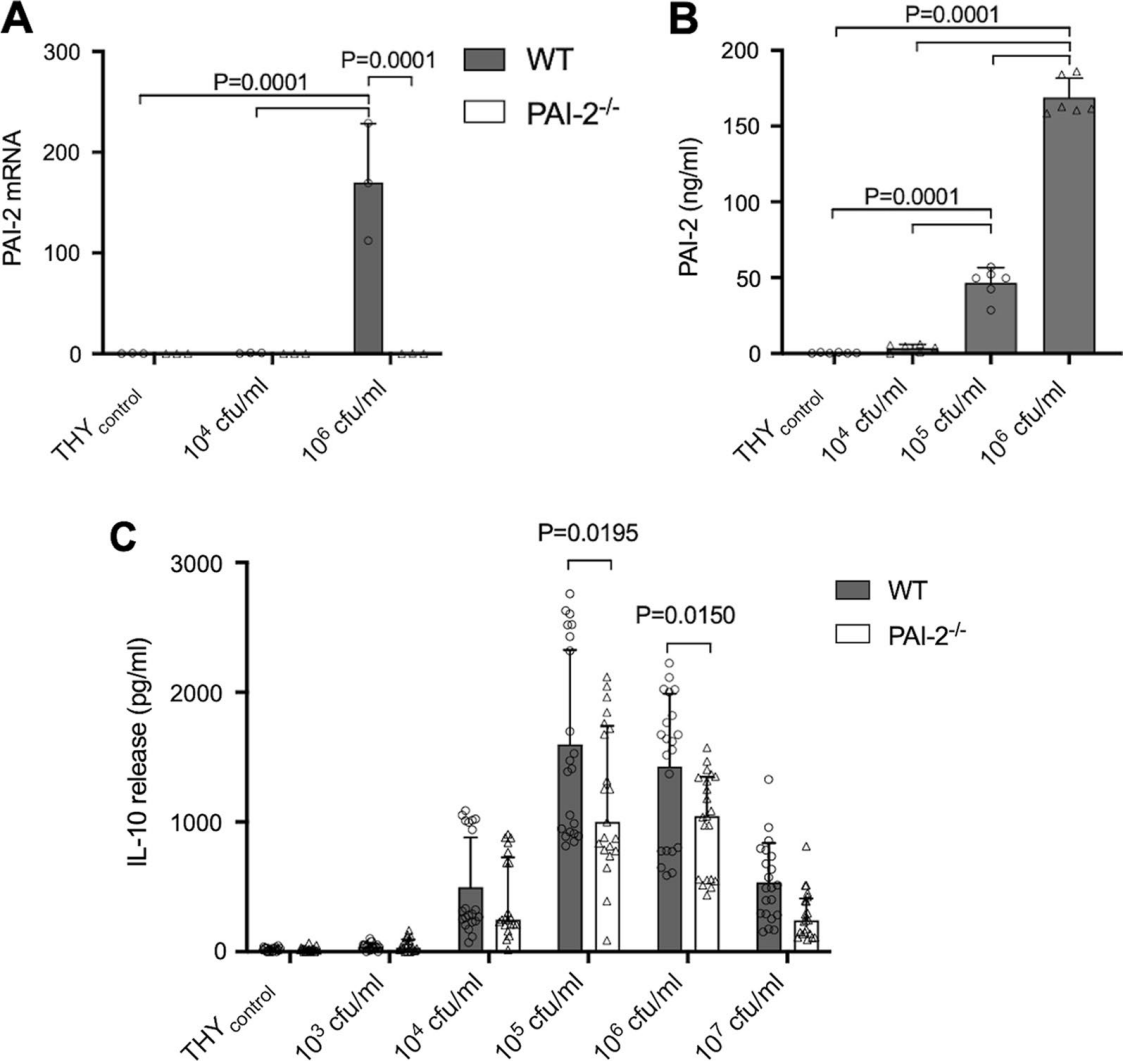
PAI-2 mRNA expression (**a**), as well as PAI-2 (**b**) and IL-10 release (**c**) into the cell culture supernatant of wt and PAI-2-deficient BMDM upon pneumococcal challenge. Pneumococcal exposure led to strong upregulation of PAI-2 mRNA expression (**a**) and increased PAI-2 release by wt BMDM (**b**). PAI-2-deficient BMDM responded to *S. pneumoniae* challenge by decreased IL-10 liberation compared to wildtype BMDM (**C**). Todd Hewith Broth with yeast (THY), culture medium for *S. pneumoniae,* was used as a control

Since macrophages express high levels of PAI-2 upon Toll-like receptor (TLR) activation [9, 17] and regulate innate immune responses to bacterial infections, we next examined the expression of PAI-2 and its functional relevance in murine BMDM upon pneumococcal challenge. Quantitative RT-PCR analysis showed a marked up-regulation of PAI-2 mRNA expression in wt BMDM, which was absent in PAI-2-deficient cells (Fig. 5a). A concentration-dependent release of PAI-2 was observed by wt BMDM into the cell culture supernatant (Fig. 5b). This liberation started at a non-cytolytic concentration of 10^5^ cfu/ml (MOI = 1) *S. pneumoniae* and increased sharply with the onset of macrophage lysis. The comparative RT-PCR analysis of selected macrophage surface and activation markers such as CD11b (ITGAM), CD206, iNOS or ARG did not reveal any differences in their expression levels between BMDM obtained from wt and PAI-2-deficient mice. There were also no differences between both genotypes in the release of the pro-inflammatory cytokines IL-1β and IL-6. However, PAI-2-deficient BMDM responded to *S. pneumoniae* challenge by a significantly decreased IL-10 liberation compared to wt BMDM (Fig. 5c). Taken together, our in vitro experiments showed a selective influence of PAI-2 deficiency on pneumococcal-induced IL-10 expression by BMDM, which is consistent with our observation in the mouse model.

## Discussion

Our results show PAI-2 expression in the inflammatory infiltrate and PAI-2 liberation into the CSF in patients and mice with PM. In mice with PM, PAI-2 deficiency was associated with increased mortality. The higher mortality was accompanied by an increase in number and size of intracerebral hemorrhages, possibly due to aggravated neutrophilic inflammation, especially around parenchymal vessels. Increased neutrophilic infiltration was paralleled by increased C5a and reduced IL-10 concentrations in brains of infected PAI-2-deficient mice, pointing to an immunoregulatory function of PAI-2 in PM. Consistent with this, murine BMDM showed increased PAI-2 production as well as reduced IL-10 release in the absence of PAI-2 after pneumococcal challenge.

PAI-2 is one of the major molecules up-regulated in monocytes and macrophages upon TLR2 and/or TLR4 engagement [9]. Accordingly, increased PAI2 expression and/or release was found in cell cultures of these cells following exposure to *Borrelia burgdorferi*, *Pseudomonas aeruginosa, Helicobacter pylori*, bacterial DNA, and meningococcal LPS-containing outer membrane vesicles [4, 20, 39, 40]. As we demonstrated in this study, infection with *S. pneumoniae* also leads to increased expression of PAI-2 and its massive release both in vitro and in vivo (animal model and patients). These observations point to a function of PAI-2 in the regulation of innate immunity. There are only a few publications in patients with sepsis [32, 35] that have addressed this hypothesis, even though mice deficient in PAI-2 have been available as a suitable tool for investigating this question for over 20 years [11]. In the initial publication, the authors showed that *Serpinb2*^−/−^mice exhibited normal development, survival, and fertility. *Serpinb2*^−/−^mice were found to exhibit no differences to wt mice in monocyte recruitment into the peritoneum after thioglycolate injection, in the degree of arterial and venous thrombosis after LPS injection into the footpad, and in survival after challenge with LPS or cecal ligation and puncture. In contrast to this negative initial phenotype analysis, it has since been reported that PAI-2-deficient mice differ from normal mice in their disease phenotype in schistosomal, lentiviral, and enteric nematode infections due to an altered TH1/TH2 immunity [27, 36, 47]. In mouse models of acute neurodegeneration, it has been shown that deficiency of PAI-2 limits brain oedema development following traumatic brain injury but did not influence lesion volume after cerebral trauma or infarction [18, 19].

In our mouse PM model, we now observed a distinct disease phenotype: PAI-2 deficiency was associated with increased mortality, which was paralleled by a substantial increase in intracerebral hemorrhages. This disease phenotype is similar to the phenotype we previously found in PAI-1-deficient (*Serpine1*^−/−^) mice [7]. Like PAI-1, PAI-2 is considered to be an authentic and physiological inhibitor of uPA and tPA within extracellular environments [1, 30]. Consequently, a lack of PAI-2 could lead to an increased formation of plasmin from plasminogen and thus to an imbalance between the coagulation and fibrinolysis system with an increased risk of bleeding. This is supported by our observations that PAI-2 deficiency was associated with increased cerebral hemorrhages in experimental PM. However, the histopathological examinations of murine brains demonstrated a substantial difference between *Serpinb2*^−/−^ and *Serpine1*^−/−^ mice. In *Serpinb2*^−/−^mice, the increase in cerebral hemorrhages was associated with pronounced neutrophilic infiltrates, while in *Serpine1*^−/−^ mice the increase in bleeding was not accompanied by enhanced inflammation around parenchymal vessels. In *Serpine1*^−/−^ mice, cerebral hemorrhage occurred also in the absence of perivascular infiltrates. This finding suggests mechanistic differences between both genotypes. In contrast to the PAI-1, PAI-2 appears to be involved in the regulation of inflammation. This is supported by our observation that *Serpinb2*^−/−^ mice had higher brain C5a levels, but lower brain IL-10 concentrations than wt mice 24 h after the start of antibiotic therapy. Previous work by our and other groups has demonstrated that C5a is a critical driver of inflammation in PM, while IL-10 appears to be an important antagonist [24, 45]. The altered expression of C5a and IL10 is likely to result in an immune imbalance in favor of a continued pro-inflammatory state. However, to what extent increased inflammation and increased fibrinolysis due to PAI2 deficiency contribute to development of cerebral hemorrhages is unclear.

How PAI-2 affects C5a and IL-10 concentrations is unclear. In myeloid cells, IL-10 production is induced by various signals downstream of pattern-recognition receptors alongside that of pro-inflammatory cytokines such as IL-1β [28, 31]. Large amounts of IL-1ß are produced and released during PM [16]. Genetic and pharmacological interference with IL-1ß generation leads to a milder immune response (and a better outcome) [22]. Recently, PAI-2 expression in macrophages has been shown to suppress IL-1β production, presumably via increasing autophagy and NLRP3 degradation, resulting in decreased caspase-1 activation [28, 31]. In our in vivo and in vitro experiments, however, PAI-2 deficiency had no impact on IL-1β production/secretion. Thus, the lower IL-10 production in the absence of PAI-2 does not appear to be related to lower IL-1β production. Consequently, the IL-10 reduction appears to be based on an IL-1beta independent mechanism that has yet to be elucidated.

The conversion of plasminogen to plasmin is tightly controlled by the availability of plasminogen activators and their inhibitors such as PAI-2. Plasmin is the only enzyme that is able to cleave complement C5 at a similar rate as the canonical C5 convertase [26], generating biological active C5a [2, 12]. Accordingly, PAI-2 deficiency could lead to increased plasmin activity and thus, to increased C5a production in PM. This pathway can contribute to a prolonged pro-inflammatory state, causing enhanced perivascular inflammation, increased cerebral hemorrhages, and reduced survival.

The analysis of CSF PAI-2 levels in patients indicating high levels of PAI-2 are associated with poor outcome are contrasting with the mouse experiments showing *absence* of PAI-2 causes worse outcome. Several explanations for this discrepancy can be provided: first, timing of CSF sampling was variable in patients, who were in different stages of the disease with variable duration and severity of symptoms, different underlying conditions, virulence of bacteria etc. Second, PAI-2 CSF levels were correlated to CSF protein levels, which indicates at least part of the PAI-2 came from leakage through the blood–brain-barrier. Finally, PAI-2 may play a role at different stages of the disease, which is difficult to extrapolate from the single measurement in CSF. Our mouse experiments show that PAI-2 deficiency is associated with increased cerebral hemorrhages, an aggravated immune response, and consequently an unfavourable clinical course. This fits well with the biological activities known so far for PAI-2.

We show *Serpinb2*^−/−^ mice have a clear disease phenotype in PM that is associated with increased perivascular neutrophilic infiltration and heavier bleeding. These observations fit with the previously discussed concepts of an immunoregulatory role of PAI-2 in pathophysiological conditions. Our study has several limitations. First, although we were able to connect the pathological changes with changes in the brain concentrations of C5a and IL-10 we were unable to clarify the underlying mechanism of these changes. Furthermore, in the animal experiments only animals surviving the full 48 h were assessed for clinical scores, weight loss, temperature, exploratory behavior, CSF white blood cell count, bacterial cerebellar titer, and brain protein cytokine concentrations. As deceased mice can be assumed to be more severely affected on most (or all) of these parameters, relevant clinical features of meningitis in PAI-2-mice may have been missed as numbers were too small to show statistically significant differences. Finally, we did not quantify fibrinolysis by measuring uPA, tPA and plasmin activity due to no (or a limited amount of) leftover CSF, blood and brain tissue. We did not feel that additional mouse experiments for this purpose only were justified.

## Conclusion

In conclusion, our data suggests that PAI-2 has an anti-inflammatory role in pneumococcal meningitis. PAI-2 deficiency increased C5a and reduced IL-10 concentration, resulting in increased mortality due to brain hemorrhages. SerpinB2 has been studied as a potential treatment in a mouse stroke model and proven to be effective in reducing brain damage [8]. Therefore, a similar therapeutic approach may be tested in a mouse PM model to assess its efficacy against meningitis-associated vascular damage and death.

## Data Availability

The datasets used and/or analyzed during the current study are available from the corresponding author on reasonable request.

## Abbreviations

BMDM: Bone marrow-derived macrophages
CFU: Colony forming units
CSF: Cerebrospinal fluid
IQR: Interquartile ranges
PAI-1: Plasminogen activator inhibitor-1
PAI-2: Plasminogen activator inhibitor-2
PBS: Phosphate buffered saline
PM: Pneumococcal meningitis
THY: Todd Hewitt Broth with yeast
TLR: Toll-like receptor
WBC: White blood cell
